# ^1^H, ^13^C and ^15^N chemical shift assignment of the stem-loops 5b + c from the 5′-UTR of SARS-CoV-2

**DOI:** 10.1007/s12104-021-10053-4

**Published:** 2022-02-18

**Authors:** Klara R. Mertinkus, J. Tassilo Grün, Nadide Altincekic, Jasleen Kaur Bains, Betül Ceylan, Jan-Peter Ferner, Lucio Frydman, Boris Fürtig, Martin Hengesbach, Katharina F. Hohmann, Daniel Hymon, Jihyun Kim, Božana Knezic, Mihajlo Novakovic, Andreas Oxenfarth, Stephen A. Peter, Nusrat S. Qureshi, Christian Richter, Tali Scherf, Andreas Schlundt, Robbin Schnieders, Harald Schwalbe, Elke Stirnal, Alexey Sudakov, Jennifer Vögele, Anna Wacker, Julia E. Weigand, Julia Wirmer-Bartoschek, Maria A. Wirtz Martin, Jens Wöhnert

**Affiliations:** 1 Institute for Organic Chemistry and Chemical Biology, Johann Wolfgang Goethe-University Frankfurt, Max-von-Laue-Str. 7, 60438 Frankfurt, Germany; 2 Institute for Molecular Biosciences, Johann Wolfgang Goethe-University Frankfurt, Max-von-Laue-Str. 7, 60438 Frankfurt, Germany; 3 Center for Biomolecular Magnetic Resonance (BMRZ), Johann Wolfgang Goethe-University Frankfurt, Max-von-Laue-Str. 7, 60438 Frankfurt, Germany; 4 Department of Chemical and Biological Physics, Weizmann Institute of Science, Herzl St. 234, 760001 Rehovot, Israel; 5 Department of Chemical Research Support, Weizmann Institute of Science, Herzl St. 234, 760001 Rehovot, Israel; 6 Present Address: EMBL Heidelberg, Meyerhofstraße 1, 69117 Heidelberg, Germany; 7 Present Address: Institute for Biochemistry, ETH Zürich, Hönggerbergring 64, 8093 Zürich, Switzerland; 8 Department of Biology, Technical University of Darmstadt, Schnittspahnstr. 10, 64287 Darmstadt, Germany; 9 Present Address: Deutero GmbH, Am Ring 29, 56288 Kastellaun, Germany

**Keywords:** SARS-CoV-2, 5′-UTR, SL5b + c, SL5b, SL5c, Solution NMR spectroscopy, COVID19-NMR

## Abstract

The ongoing pandemic of the respiratory disease COVID-19 is caused by the SARS-CoV-2 (SCoV2) virus. SCoV2 is a member of the Betacoronavirus genus. The 30 kb positive sense, single stranded RNA genome of SCoV2 features 5′- and 3′-genomic ends that are highly conserved among Betacoronaviruses. These genomic ends contain structured *cis*-acting RNA elements, which are involved in the regulation of viral replication and translation. Structural information about these potential antiviral drug targets supports the development of novel classes of therapeutics against COVID-19. The highly conserved branched stem-loop 5 (SL5) found within the 5′-untranslated region (5′-UTR) consists of a basal stem and three stem-loops, namely SL5a, SL5b and SL5c. Both, SL5a and SL5b feature a 5′-UUUCGU-3′ hexaloop that is also found among Alphacoronaviruses. Here, we report the extensive ^1^H, ^13^C and ^15^N resonance assignment of the 37 nucleotides (nts) long sequence spanning SL5b and SL5c (SL5b + c), as basis for further in-depth structural studies by solution NMR spectroscopy.

**Supplementary Information:**

The online version contains supplementary material available at 10.1007/s12104-021-10053-4.

## Biological context

The ongoing global pandemic associated with the coronavirus disease (COVID-19) is caused by the human Betacoronavirus SARS-CoV-2 (SCoV2), a close relative of the severe acute respiratory syndrome (SARS) causing agent SARS-CoV. Betacoronaviruses have large positive sense, single-stranded RNA genomes, with highly conserved 5′- and 3′-untranslated regions (UTRs) that do not code for viral proteins. These structured UTRs are highly conserved among Betacoronaviruses and are important for the replication, balanced transcription of subgenomic mRNAs and translation of viral proteins. (Yang and Leibowitz 2015) So far, most efforts for the development of new antiviral drugs target the proteins of SARS-CoV-2. The structured regulatory elements of the approx. 30,000 nucleotides (nts) long RNA genome remain unexploited as potential target sites for antiviral drugs. Between different Coronaviruses, the sequence of the individual elements varies, but their secondary structures reveal remarkably high conservation, suggesting a critical importance for viral viability and pathogenesis. (Madhugiri et al. 2016) Until now, a large number of sequence-based computational predictions and different chemical probing approaches have been reported to map the architecture of these viral RNA elements. (Zhao et al. 2020; Huston et al. 2021; Lan et al. 2021; Manfredonia and Incarnato 2021; Rangan et al. 2021) However, to establish the viral RNA as an antiviral drug target, high-resolution structural data are important that can also visualize structural dynamics and tertiary structure interactions. In response to the pandemic situation, the international COVID19-NMR initiative (https://covid19-NMR.de) has set the goal to provide this information by solution NMR, in order to initiate and guide structure-based drug screening, design and synthesis. The structured parts of the SARS-CoV-2 genome have been divided into fragments in a ‘divide and conquer’ approach, allowing us to determine the secondary structures of these RNA elements. (Wacker et al. 2020) Further, fragment screening campaigns demonstrated that the RNA structural elements can be targeted differentially, revealing low micromolar binding affinities specific to molecules of low molecular weight. (Sreeramulu et al. 2021).

An intriguing example of an RNA regulatory element from SARS-CoV-2 is the comparably large structural element of SL5 spanning nts 149–265. The entire SL5 element consists of four helices, joining three sub-elements with stem loop motifs to the SL5 basal stem by a four-way junction. These sub-elements are termed SLs 5a, 5b and 5c. Interestingly, SL5 is forming junction-connected elements in the genomes of both Alpha- and Betacoronaviruses. (Madhugiri et al. 2014, 2016) The regulatory function of SL5 has been linked to maintaining efficient viral replication. (Chen and Olsthoorn 2010; Guan et al. 2011) In SL5b, an apical hexaloop sequence is found that is identical to the loop in SL5a (5'-UUUCGU-3′). Similar loop sequences with 5′-UUYCGU-3′ motifs can also be found in members of the Alphacoronavirus genus, suggesting a conserved function e.g. in viral packaging. (Masters 2019) Interestingly, currently available sequencing data for new SCoV-2 variants emerging since March 2020 show that the 5′-UUUCGU-3′ loop in SL5a remains conserved compared to the original virus strain, while a C241U mutation resulting in a 5′-UUU**U**GU-3′ loop appeared in SL5b.

**Fig. 1:**
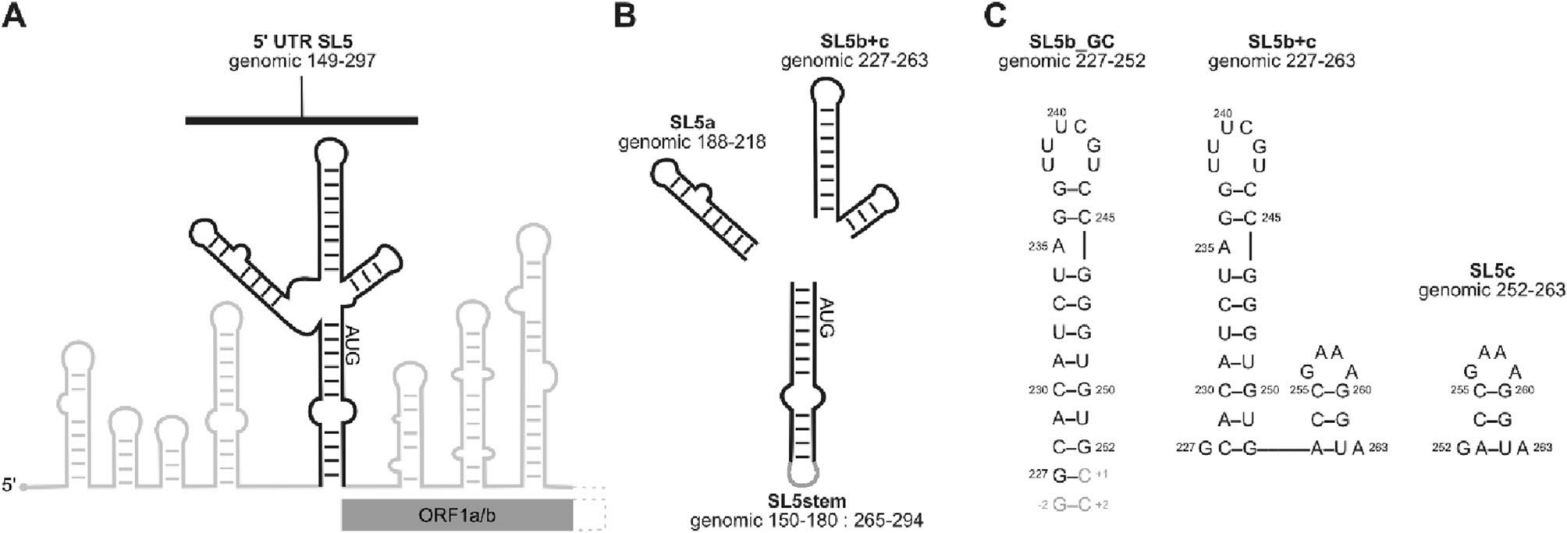
**A** Schematic overview of 5′-UTR RNA elements of the SCoV2 genome. Black: SL5 element; AUG start codon and the 5′-terminal structural elements of the open reading frame ORF1a/b are highlighted in grey. **B** Elements used for the NMR-based divide-and-conquer approach. **C** Predicted secondary structures of RNA (sub-)elements used for the NMR chemical shift assignment of SL5b + c reported here. Genomic region, numbering and sample titles are given. **B/C** Black regions according to genomic sequence, grey regions contain stabilizing nucleotides. The actual investigated RNAs are represented by the sequences including the grey regions

In SCoV2, SL5 contains the first 29 nts of the open reading frame ORF 1a/b that codes for nsp1, the first of the non-structural proteins (Fig. 1) including the start codon A266 to G268, suggesting that the complex structural arrangement in SL5 is important for translation initiation. The SL5b stem-loop contains nucleotides 228 to 252 (25 nts), while the downstream located SL5c consists of 10 nts, 253 to 262. We report here the NMR chemical shift assignments for SL5b + c (nts 227–263) containing both stems, which was aided by assigning the isolated SL elements based on initial ^1^H and ^15^N assignments of all sub-elements of SL5 (a–c) and the basal stem. (Wacker et al. 2020) More recently, we reported the chemical shift assignments including ^13^C chemical shifts for SL5a. (Schnieders et al. 2021).

## Methods and experiments

### Sample preparation

RNA synthesis for NMR experiments: For DNA template production, the sequences of SL5b + c (genomic nucleotides 227 to 263) and SL5b_GC, (5′-G-(genomic 227 to 252)-CC-3′) (Fig. 1C), together with the T7 promoter were generated by hybridization of complementary oligonucleotides and introduced into the *EcoR*I and *Nco*I sites of a plasmid, based on the pSP64 vector (Promega) encoding an HDV ribozyme (Schürer et al. 2002). RNAs were transcribed as HDV ribozyme fusions to obtain homogeneous 3′-ends. The recombinant vectors pHDV-5_SL5b + c and pHDV-5_SL5b_GC were transformed and amplified in the *Escherichia coli* strain DH5α. Plasmid-DNA was purified with a large scale DNA isolation kit (Gigaprep; Qiagen) according to the manufacturer’s instructions and linearized with *Hind*III prior to *in-vitro* transcription by T7 RNA polymerase [P266L mutant, prepared as described in (Guillerez et al. 2005; Schnieders et al. 2020)]. 15 ml transcription reactions [20 mM DTT, 2 mM spermidine, 200 ng/µl template, 200 mM Tris/glutamate (pH 8.1), 40 mM Mg(OAc)_2_, 12 mM NTPs, 32 µg/ml T7 RNA Polymerase, 20% DMSO (b + c) or 0% DMSO (b_GC)] were performed to obtain sufficient amount of RNA. Preparative transcription reactions (6 h at 37 °C and 70 rpm) were terminated by addition of 150 mM EDTA. SL5b + c and 5SL5b_GC RNAs were purified as follows: RNAs were precipitated with one volume of 2-propanol at − 20 °C overnight. RNA fragments were separated on 12–15% denaturing polyacrylamide (PAA) gels and visualized by UV shadowing at 254 nm. SL5b + c and SL5b_GC containing RNA bands were excised from the gel and then incubated for 30 min at − 80 °C, followed by 15 min at 65 °C. The elution was done overnight by passive diffusion into 0.3 M NaOAc, precipitated with EtOH and desalted via PD10 columns (GE Healthcare). Residual PAA was removed by reversed-phase HPLC using a Kromasil RP 18 column and a gradient of 0–40% 0.1 M triethylammonium acetate in acetonitrile. After freeze-drying of RNA-containing fractions and cation exchange by LiClO_4_ precipitation (2% in acetone), the RNA was folded in water by heating to 80 °C followed by rapid cooling on ice. Buffer exchange into NMR buffer (95% H_2_O/5% D_2_O, 25 mM potassium phosphate buffer, pH 6.2, 50 mM potassium chloride) was performed using Vivaspin centrifugal concentrators (2 kDa molecular weight cut-off). The purity of SL5b + c and SL5b_GC was verified by denaturing PAA gel electrophoresis and homogeneous folding was monitored by native PAA gel electrophoresis, loading the same RNA concentration as used in NMR experiments. 100% D_2_O samples were prepared by lyophilisation and redissolving in identical volumes of pure D_2_O to keep the buffer salt concentration constant.

Using this protocol, three NMR samples of SL5b + c were prepared: a 350 µM uniformly ^15^N- and two uniformly ^13^C, ^15^N-labelled samples (750 µM in buffer with 95% H_2_O/5% D_2_O and 430 µM in buffer with 100% D_2_O). In addition, two uniformly ^13^C, ^15^N-labelled samples of SL5b_GC were prepared: an H_2_O sample at a concentration of 510 µM in buffer with 95% H_2_O/5% D_2_O and a D_2_O sample at a concentration of 300 µM in buffer with 100% D_2_O. For the divide-and-conquer approach, the unlabelled single stem-loop 5c (Fig. 1C) was purchased from Horizon Discovery LTD (Cambridge, UK). The sample was processed by reversed-phase HPLC identical to the in-vitro transcribed samples. LiClO_4_ precipitation, buffer exchange, folding check and sample preparation was performed as mentioned above (1.1 mM in NMR buffer with 95% H_2_O/5% D_2_O, 1.0 mM in NMR buffer with 100% D_2_O).

### NMR experiments

NMR experiments were carried out at the Weizmann Institute (WIS) using a Bruker AVIII 600 MHz NMR spectrometer equipped with a 5 mm, z-axis gradient ^1^H [^13^C, ^15^N]-TCI prodigy probe and a Bruker AVANCE Neo 1 GHz spectrometer equipped with a 5 mm, z-axis gradient ^1^H [^13^C, ^15^N]-TCI cryogenic probe and at the Center for Biomolecular Magnetic Resonance (BMRZ) at the Goethe University Frankfurt using Bruker NMR spectrometers from 600 to 800 MHz, which are equipped with AVANCE Neo, AVIIIHD, AVIII and AVI consoles and the following cryogenic probes: 5 mm, z-axis gradient ^1^H [^13^C, ^31^P]-TCI cryogenic probe (600 MHz), 5 mm, z-axis gradient ^1^H/^19^F [^13^C, ^15^N]-TCI prodigy probe (600 MHz), 5 mm, z-axis gradient ^1^H [^13^C, ^15^N]-TCI cryogenic probe (600 MHz), 5 mm, z-axis gradient ^1^H [^13^C, ^15^N,^31^P]-QCI cryogenic probe (700 MHz) and ^13^C-optimized 5 mm, z-axis gradient ^13^C [^15^N, ^1^H]-TXO cryogenic probe (800 MHz).

Experiments were performed in a temperature range spanning 274 to 298 K. NMR spectra were processed and analysed using Topspin (versions 3.6.2 to 4.1.1), and chemical shift assignment was conducted using Sparky. (Lee et al. 2015) NMR data were managed and archived using the platform LOGS (2020, version 2.1.54, Signals GmbH & Co KG, www.logs.repository.com). ^1^H chemical shifts were referenced externally to DSS and ^13^C and ^15^N chemical shifts were indirectly referenced from the ^1^H chemical shift as previously described. (Wishart et al. 1995).

## Assignment and data deposition

The imino, aromatic and ribose resonances of the SL5b + c were assigned using a ^13^C, ^15^N-labelled SL5b_GC sample and an unlabelled SL5c model RNA (SL5c) (SI Fig. 1).

**Fig. 2:**
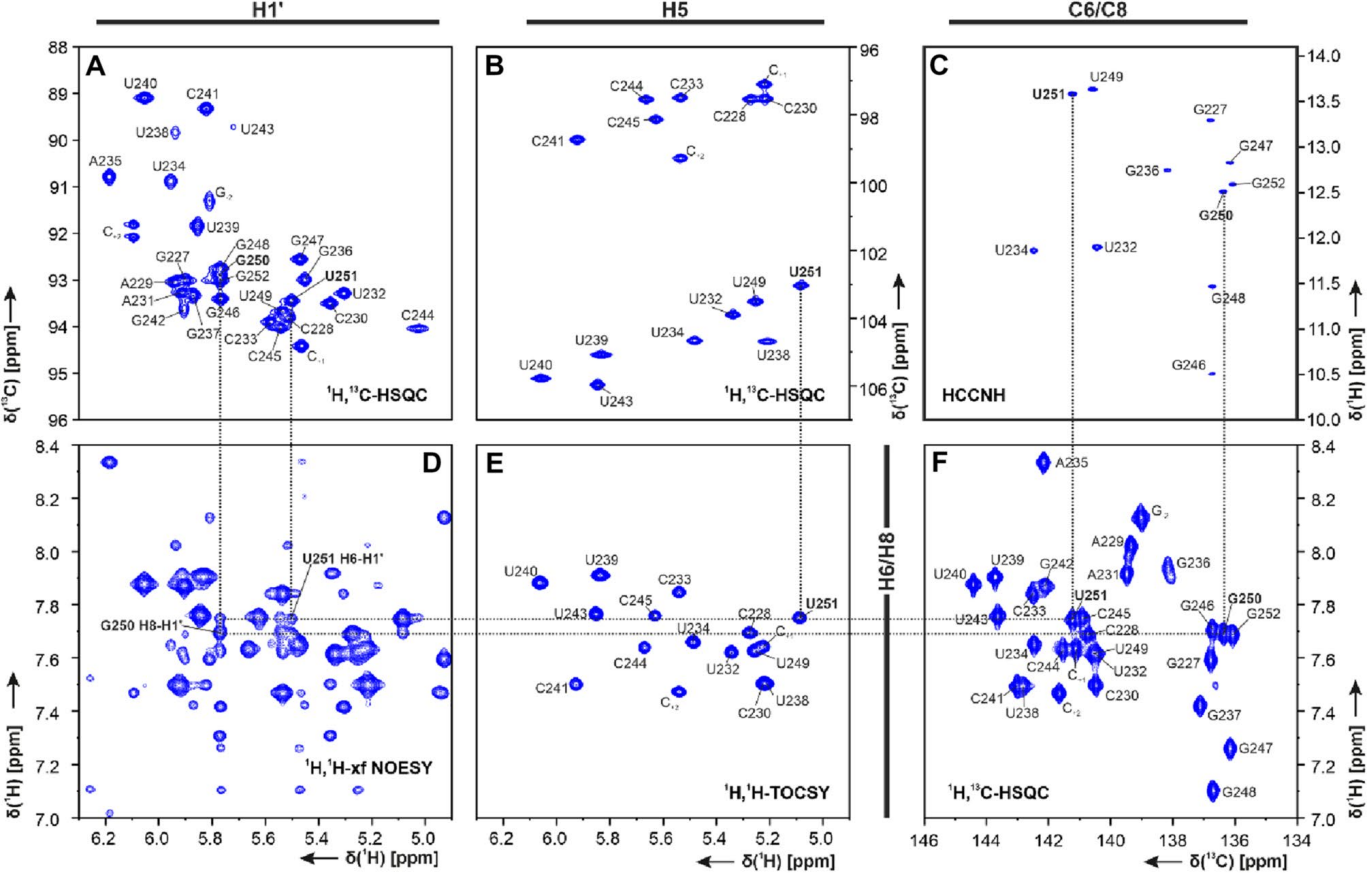
Spectra of SL5b_GC, in NMR buffer in 95% H_2_O/5% D_2_O, 298 K: **A** ^1^H,^13^C-HSQC (C1′–H1′ region), **B** ^1^H,^13^C-HSQC (C5–H5 region), **C** HCCNH, **D** ^1^H,^1^H-xfilter NOESY, **E** ^1^H,^1^H-TOCSY and **F** ^1^H,^13^C-HSQC (C6–H6/C8–H8 region). Annotation of nucleobase assignment uses genomic numbering. Additional closing base pairs are annotated with ‘± x’. Dashed lines showing examples of ribose-to-aromatic atom relations for bases G250 and U251 of the helical region. (For experimental details see SI Table 2)

**Fig. 3:**
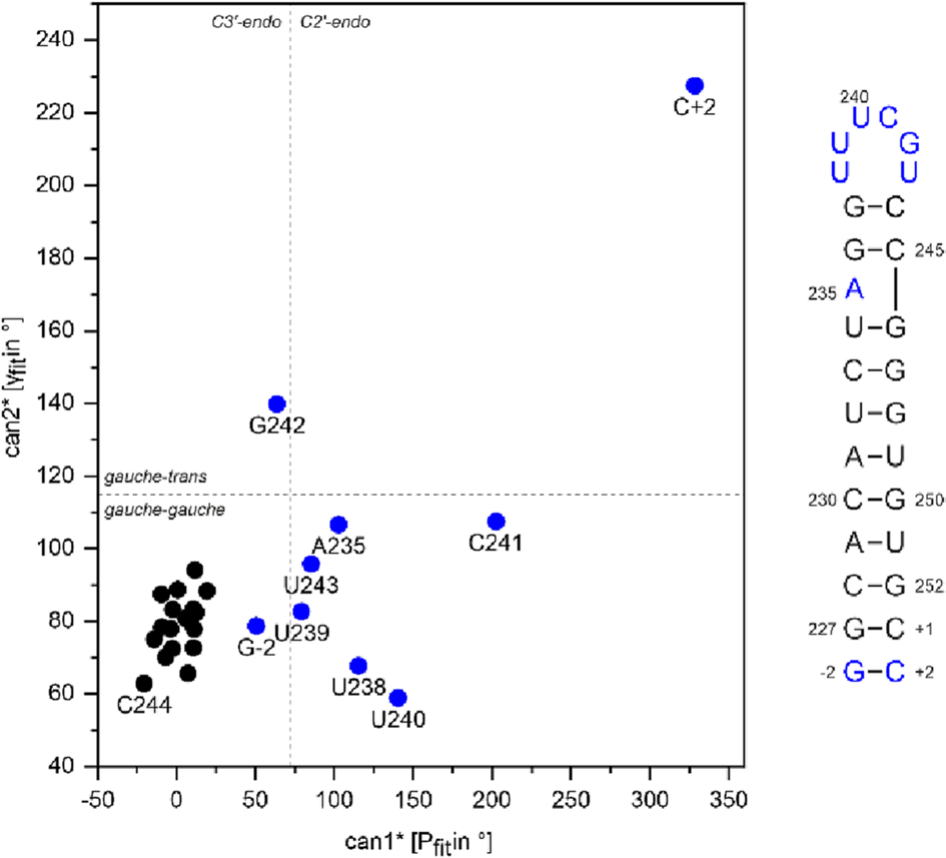
Graph of canonical coordinates can1*[Pfit in °] and can2*[γfit in °] for SL5b_GC, calculated as in (Cherepanov et al. 2010). Data points are annotated by base numbering as used in the RNA secondary structure scheme on the right. Blue (both in the graph and the secondary structure) highlights residues with non-C3′-endo conformation or deviations in exocyclic torsion angle γ

The assignment of SL5b_GC is described in the following using the experiments summarized in SI Table SL5b_GC. From the imino proton chemical shift assignment by ^1^H, ^15^N-TROSY (SI Fig. 2A), ^1^H,^1^H-NOESY (SI Fig. 2B) and HNN-COSY (SI Fig. 2C) spectra, the U-C2 and -C4 as well as G-C2 and -C6 could be assigned in the ^1^H, ^13^C-HNCO, and as well for aromatic carbon resonances U-H3/C6 and G-H1/C8 in the ^1^H, ^13^C-HCCNH (Fig. 2C). Using an ^1^H,^1^H-TOCSY (Fig. 2E) to selectively assign cytidine and uridine H5-H6 resonances and ^1^H, ^13^C-HSQC's (Fig. 2A, B, and F) for aromatic carbon resonances, the pyrimidine base C5-H5 and C6-H6 were obtained. Assignment of purine C8-H8 was aided by an ^1^H,^1^H-x-filter-NOESY (Fig. 2D). The latter experiment was also used to confirm assigned shifts for aromatic H6/H8 and H1′ found in a 3D-NOESY-HSQC and 3D-HCN. Additional carbons C4, C5 and C6 for adenosine were assigned using a 3D TROSY-HCCH-COSY. Nucleobase intra and sequential aromatic-to-ribose H1′ correlations were successfully detected for the predicted helical stem part from G-2 to U238 and from U243 to C + 2. The H1′ assignments were confirmed by a 3D-NOESY-HSQC leading to almost complete H1′ assignments with the help of a ^1^H,^13^C-HSQC for the H1′–C1′ region (Fig. 2A). From here, a ^1^H,^13^C-ct-HSQC and 3D-HCCH-TOCSY's with different mixing times gave further insight to the CH ribose resonance shifts. A canonical shift analysis for the sugar puckers of the almost completely assigned C1′ to C5′ resonances showed a C3′-endo conformation except for the bulge and loop nucleotides (Fig. 3**)**. Additionally, chemical shift assignments for the nitrogens N1 or N9 could be assigned in the ^1^H,^15^N-HCN experiment.

The assignments obtained for the SL5b_GC sample were transferred to ^1^H,^15^N-TROSY, ^1^H,^1^H-NOESY, ^1^H,^13^C-HNCO and ^1^H,^13^C-HSQC spectra of SL5b + c (Table 1I to IV), showing a fit of the shifts of SL5b_GC from nucleotides ranging from C230 to G250. Small chemical shift differences are in line with the differences in primary chemical structure.

**Table 1:** List of NMR experiments for SL5b + c conducted at WIS and BMRZ at temperatures a: 275 K, b: 283 K and c: 298 K. Spectra were recorded in NMR buffer with A: 95% H_2_O/5% D_2_O or B: 100% D_2_O. Experimental parameters and experiment-specific parameters are given

#	NMR experiment	Experimental parameters	Characteristic parameters
I	^1^H,^15^N-TROSY^BMRZ^	**A** a,b: 700 MHz, ns: 16, sw(f2): 22.04 ppm, sw(f1): 25.62 ppm, aq(f2): 70.4 ms, aq(f1): 64.9 ms, o1(^1^H): 4.7 ppm, o2(^13^C): 101 ppm, o3(^15^N): 153 ppm, rel. delay: 0.3 s, time: 30 min **A** c: 700 MHz, ns: 16, sw(f2): 21.0 ppm, sw(f1): 25.3 ppm, aq(f2): 71.3 ms, aq(f1): 69.6 ms, o1(^1^H): 4.7 ppm, o2(^13^C): 101 ppm, o3(^15^N): 153 ppm, rel. delay: 0.3 s, time: 30 min	
II	^1^H,^1^H-NOESY^WIS^ jump-return water suppression	**A** a imino: 600 MHz, ns: 32, sw(f2): 22.03 ppm, sw(f1): 12.00 ppm, aq(f2): 77.4 ms, aq(f1): 53.3 ms, o1(^1^H): 4.692 ppm, o3(^15^N): 155 ppm, rel. delay: 2.0 s, time: 11 h 30 min **A** b imino: 600 MHz, ns: 40, sw(f2): 20.83 ppm, sw(f1): 11.49 ppm, aq(f2): 81.9 ms, aq(f1): 37.1 ms, o1(^1^H): 4.7 ppm, o3(^15^N): 153 ppm, rel. delay: 1.5 s, time: 10 h	**A**: NOE mixing time 150 ms, JR-delay 200 µs
III	^1^H,^15^N-cmpg-NOESY^WIS^ ^imino and amino cross−correlations^	**A** b 600 MHz, ns: 96, sw(f2): 20.83 ppm, sw(f1): 102.72 ppm, aq(f2): 81.9 ms, aq(f1): 20.4 ms, o1(^1^H): 4.7 ppm, o2(^13^C): 101 ppm, o3(^15^N): 117 ppm, rel. delay: 2.0 s, time: 15 h 30 min	
IV	^1^H,^13^C-HSQC^WIS^ aromatic region (Bodenhausen and Ruben 1980)	**A** b: 600 MHz, ns: 16, sw(f2): 9.80 ppm, sw(f1): 26.00 ppm, aq(f2): 87.04 ms, aq(f1): 16.3 ms, o1(^1^H): 4.7 ppm, o2(^13^C): 143 ppm, o3(^15^N): 153 ppm, rel. delay: 1.0 s, time: 40 min **A** c: 600 MHz, ns: 16, sw(f2): 9.80 ppm, sw(f1): 26 ppm, aq(f2): 87.04 ms, aq(f1): 16.3098 ms, o1(^1^H): 4.7 ppm, o2(^13^C): 143 ppm, o3(^15^N): 153 ppm, rel. delay: 1.0 s, time: 40 min	INEPT transfer time 2.7 ms (^1^J_CH_ 185 Hz), off-resonant Q3 shaped pulse for C5 decoupling at 95 ppm with 25 ppm bandwidth
III	^1^H,^13^C-HSQC^BMRZ^ full	**A** b 800 MHz, ns: 8, sw(f2): 10.0 ppm, sw(f1): 110.4 ppm, aq(f2): 64.0 ms, aq(f1): 11.5 ms, o1(^1^H): 4.7 ppm, o2(^13^C): 105 ppm, o3(^15^N): 153 ppm, rel. delay: 1.0 s, time: 1 h 15 min	HSQC with gradient selection, INEPT transfer time 1.6 ms (^1^J_CH_ 160 Hz)
IV	^1^H,^13^C-ct-HSQC^BMRZ^ ribose region C1’ to C5′	**B** c 900 MHz, ns: 4, sw(f2): 8.29 ppm, sw(f1): 38.0 ppm, aq(f2): 68.6 ms, aq(f1): 14.8 ms, o1(^1^H): 4.7 ppm, o2(^13^C): 77 ppm, o3(^15^N): 150 ppm, rel. delay: 1.0 s, time: 20 min	INEPT transfer time 1.6 ms (^1^J_CH_ 160 Hz), CT period 12.5 ms (^1^J_CC_ 77 Hz)
V	(H)C(CCN)H^WIS^ imino-to-aromatics (Piotto et al. 1992; Sklenář et al. 1996)	**A** b 600 MHz, ns: 256, sw(f2): 20.8 ppm, sw(f1): 9.94 ppm, aq(f2): 81.9 ms, aq(f1): 42.6 ms, o1(^1^H): 4.7 ppm, o2(^13^C): 137 ppm, o3(^15^N): 154 ppm, rel. delay: 1.5 s, time: 16 h **A** c 600 MHz, ns: 208, sw(f2): 20.8 ppm, sw(f1): 9.94 ppm, aq(f2): 90.2 ms, aq(f1): 42.6 ms, o1(^1^H): 4.7 ppm, o2(^13^C): 137 ppm, o3(^15^N): 154 ppm, rel. delay: 1.8 s, time: 15 h	CC-TOCSY mixing time 28 ms
VI	H(N)CO^BRMZ,WIS^ imino-to-carbon (Favier and Brutscher 2011; Solyom et al. 2013)	**A** a 700 MHz, ns: 128, sw(f2): 20.8 ppm, sw(f1): 31.0 ppm, aq(f2): 60.3 ms, aq(f1): 23.4 ms, o1(^1^H): 4.7 ppm, o2(^13^C): 153 ppm, o3(^15^N): 158 ppm, rel. delay: 0.3 s, time: 4 h 15 min **A** b 1000 MHz, ns: 64, sw(f2): 21.7 ppm, sw(f1): 22.0 ppm, aq(f2): 94.2 ms, aq(f1): 11.6 ms, o1(^1^H): 4.7 ppm, o2(^13^C): 153 ppm, o3(^15^N): 159 ppm, rel. delay: 0.5 s, time: 9 h	
VII	3D ^1^H,^13^C-NOESY-HSQC^WIS^ aromatics (Piotto et al. 1992; Sklenáŕ et al. 1993)	**A** b,c 600 MHz, ns: 16, sw(f3,^1^H): 9.8 ppm, sw(f2,^13^C): 21.0 ppm, sw(f1,^1^H): 14.4 ppm, aq(f3): 87.0 ms, aq(f2): 10.1 ms, aq(f1): 8.33 ms, o1(^1^H): 4.7 ppm, o2(^13^C): 142 ppm, o3(15N): 154 ppm, rel. delay: 1.0 s, time: 2 d 7 h	NOE mixing time 200 ms
VIII	3D HCCH-TOCSY^BMRZ^ ribose C1′-to-C2′ (Kay et al. 1993; Richter et al. 2010)	**B** c 800 MHz, ns: 8, sw(f3,^1^H): 8.5 ppm, sw(f2,^13^C): 9.5 ppm, sw(f1,^13^C): 35.5 ppm, aq(f3): 74.7 ms, aq(f2): 21 ms, aq(f1): 11.2 ms, o1(^1^H): 4.7 ppm, o2(^13^C): 76.5 ppm, o3(^13^C): 76.5 ppm, rel. delay: 1.0 s, time: 1 d 10 h	CC-TOCSY mixing time 6 ms
IX	3D HCCH-TOCSY^BMRZ^ ribose C1′-to-C5′ (Kay et al. 1993; Richter et al. 2010)	**B** c 800 MHz, ns: 8, sw(f3,^1^H): 8.5 ppm, sw(f2,^13^C): 9.5 ppm, sw(f1,^13^C): 35.5 ppm, aq(f3): 74.7 ms, aq(f2): 21 ms, aq(f1): 11.2 ms, o1(^1^H): 4.7 ppm, o2(^13^C): 76.5 ppm, o3(^13^C): 76.5 ppm, rel. delay: 1.0 s, time: 1 day 10 h	CC-TOCSY mixing time 18 ms

*ns* number of scans, *sw* spectral width, *aq* acquisition time, *o1/2/3* carrier frequencies on channels *1/2/3, rel. delay* relaxation delay, *CT* constant time, *JR* jump-return

**Fig. 4:**
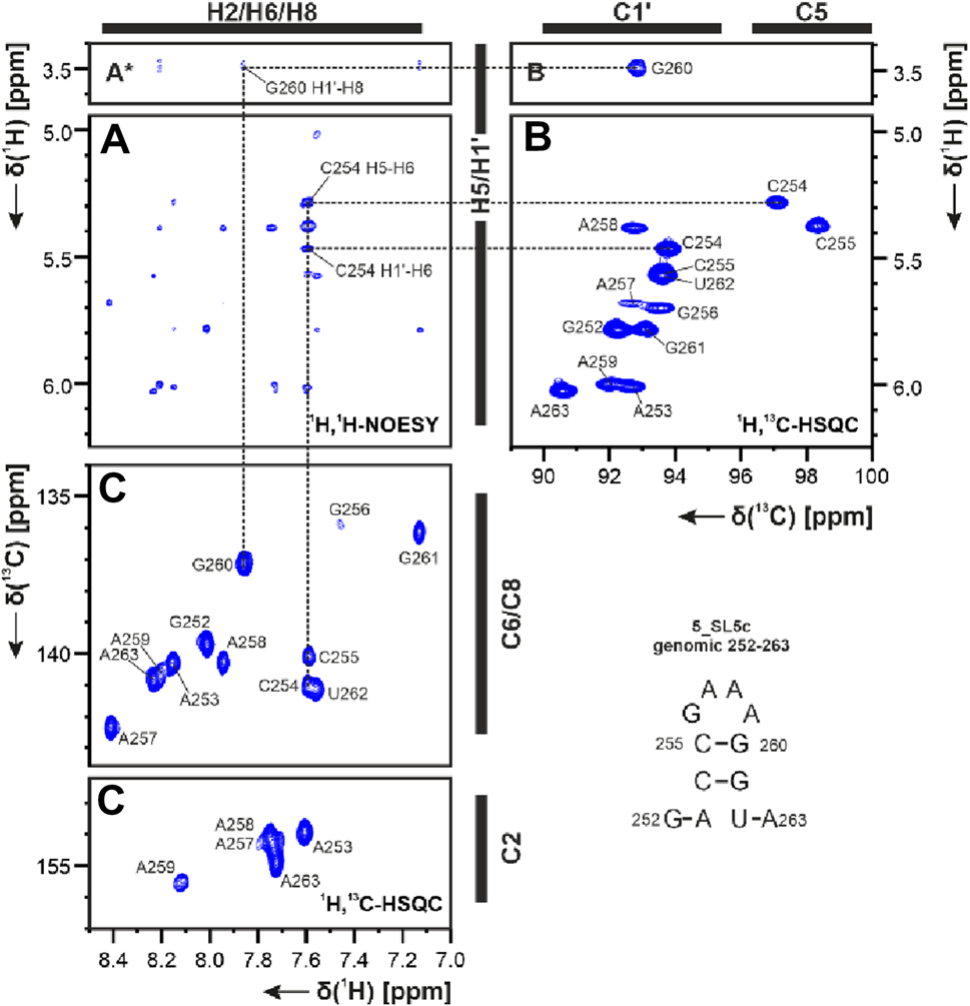
**A** ^1^H,^1^H-NOESY, **B** ^1^H,^13^C-HSQC (C1’ region) and **C** ^1^H,^13^C-HSQC (aromatic region) spectra for aromatic and ribose resonances of SL5c at 283 K in NMR buffer with 100% D_2_O. *Lower contour level setting. Exemplary correlations are annotated by dashed lines and using the genomic numbering (for experimental details see SI Table 2)

The assigned imino resonances of the SL5c sample provided a starting point for the aromatic proton resonances assignment of the ^1^H,^1^H-NOESY experiment (for experimental data see SI Table 2). The sequential walk was only interrupted by missing cross peaks between G256-H8 to A257-H8 in the loop region in both the 95% H_2_O and the D_2_O (Fig. 4A) samples in NMR buffer. Using ^1^H,^1^H-TOCSY and ^1^H,^13^C-HSQC (Fig. 4B and C) the aromatic resonances of protons H2, H6, H8 and H5 as well as their corresponding carbons except for U262-C5 were obtained. Using a ^1^H,^1^H-NOESY recorded for a sample diluted in D_2_O (Fig. 4A), the H1′,C1′ ribose resonances were assigned by the analysis of intra-nucleotide and sequential NOEs. Similar to this, the H2′–C2′ assignments were obtained by identification of H1′–H2′ intra- nucleotide and sequential NOEs.

### The 5′-UUUCGU-3′ hexaloop in SL5b

The entire SL5 motif **(**Fig. 1A) spans three stem-loop elements. Interestingly, SL5a and SL5b both possess the same 5′-UUUCGU-3′ hexaloop consisting of nucleotides 238–243 (SL5a: 200–205). While in SL5a, the loop closing stem is formed by at least three base pairs, the hexaloop of SL5b is closed by a stem consisting of only two GC base pairs, preceded by a bulge at residue A235. This bulged A235 shows downfield shifted signals for the aromatic H2 and H8 in the aromatic ^1^H,^13^C-HSQC, as typically observed for non-stacked purines. (Aeschbacher et al. 2013) Starting from H8 of residue G237, assignment of the ribose H1′ and aromatic H6/H8 was obtained by a sequential walk obtained in an amino nitrogen filtered NOESY (x-filter-NOESY). Loop residues U240 and C241 show characteristic C1′–H1′ shifts in the ^1^H,^13^C-HSQC, which reveal a fingerprint characteristic for the hexaloop. The overall resonance assignment of the hexaloop is in excellent agreement with the observations in SL5a. This loop arrangement is similar to a UUCG-tetraloop, for which detailed structural restraints are available. (Fürtig et al. 2004; Nozinovic et al. 2010).

Remarkably, available sequencing data for observed mutations in SARS-CoV2 (Hadfield et al. 2018; Cao et al. 2021), show significantly different vulnerability for mutations for the two hexaloop sequences. While the SL5a loop sequence remained mostly conserved until recently, in SL5b mutation C241U appeared in variants emerging since March 2020. A first study on mutational frequency indicates, among others, high C-to-U mutation rates in the SCoV2 genome. (Mourier et al. 2021) With the most recent mutation at SL5a C203U, both hexaloop sequences change to 5′-UUUUGU-3′. With the chemical shifts provided here, the delineation of structural differences for mutant versions of SCoV2 from changes in chemical shifts can be monitored by NMR spectroscopy.

### The GAAA-tetraloop of SL5c

The GAAA-tetraloop of the SL5c stem consists of G256 to A259, closed by two GC and one AU base pairs. All three G N1–H1 resonances observed for the shorter construct were superimposable in the ^1^H,^15^N TROSY spectrum of SL5b + c. The chemical shifts observed in SL5b + c are in agreement with chemical shifts for a GAAA tetraloop (Jucker et al. 1996). Particularly, the ribose H1′ shift of ~ 3.5 ppm of the guanosine residue in the loop closing base pairing is characteristic. ^31^P 1D data support the formation of a typical GAAA-tetraloop (SI Fig. 3, (Legault and Pardi 1994)). Legault and Pardi detected shows a stabilization of the G imino ^1^H (corresponding to G256 in our construct) by interaction with a phosphate oxygen in the backbone for a GAAA tetraloop. While we could not assign crosspeaks between the loop guanosine (G256) and the loop adenosine (A259) that would have confirmed the reported loop geometry in SL5c, we found an additional imino crosspeak assigned to G256. In addition to the imino and amino proton assignment, which is consistent with the published chemical shifts for SL5b + c (Wacker et al. 2020), complete assignments of the aromatic C–H resonances as well as the ribose resonances C1′–H1′ and C2′–H2′ were obtained for element SL5c. While in SL5c imino signals of base pairs were observable at 298 K, a vanishing of these signals as well as appearance of additional signals and shifting of signals in the aromatic region of SL5c was noticed within the larger SL5b + c context. Thus, the SL5c stem is more stable and opens only at higher temperatures in the full-length construct (SI Fig. 4).

## Summary

We herein present the ^1^H, ^13^C, ^15^N chemical shifts of SL5b + c, using two fragments, SL5b_GC and SL5c, that subsequently allowed assignment of the SL5b + c element. The assignments of the sub-constructs were used as starting points for advancing the SL5b + c assignment. For the combined construct, an overall assignment was conducted at temperatures of 274 to 298 K. 95% of the aromatic H6–C6 and H8–C8 resonances were assigned for SL5b + c as well as the 8 adenosine H2–C2, and 17 uridine and cytidine H5–C5. Carbonyl and other quaternary carbon atoms of the nucleobases were partly assigned: in purines (C2: 75%, C4: 15% and C6: 50%) and pyrimidines (C2: 29% and C4: 24%). The assignment of the nitrogen atoms includes 70% N1 for purines and 65% N3 for pyrimidines, which represent mostly those involved in hydrogen bonding interactions. With the assignment transfer 90% of H1′–C1′ chemical shift assignment was obtained. Further ribose resonances for H2′ to H5″ and C2′ to C5′ are partially assigned in ranges of 10 to 30%. In summary, an assignment of 65% of the ^1^H, 53% of the ^13^C and 63% of the ^15^N atoms in the nucleobases of SL5b + c has been achieved.

## Supplementary Information

Below is the link to the electronic supplementary material.

Supplementary file1 (DOCX 500 KB)

## Data Availability

We updated the BMRB deposition for SL5b + c with code 50339. Data of SL5b_GC are found in BMRB code 51138 and SL5c BRMB code 51137.
