# Supplementary material for: 1H, 13C and 15N chemical shift assignment of the stem-loops 5b + c from the 5′-UTR of SARS-CoV-2
Source: Biomol NMR Assign. 2022 Feb 18;16(1):17–25. doi: 10.1007/s12104-021-10053-4 (PMC8853908; doi:10.1007/s12104-021-10053-4)
Supplement: Supplementary file 1 — Supplementary file1 (DOCX 500 KB) [file 12104_2021_10053_MOESM1_ESM.docx]

**Supporting Information**

**^1^H, ^13^C and ^15^N chemical shift assignment of the stem-loops 5b + c from the 5'-UTR of SARS-CoV-2**

**
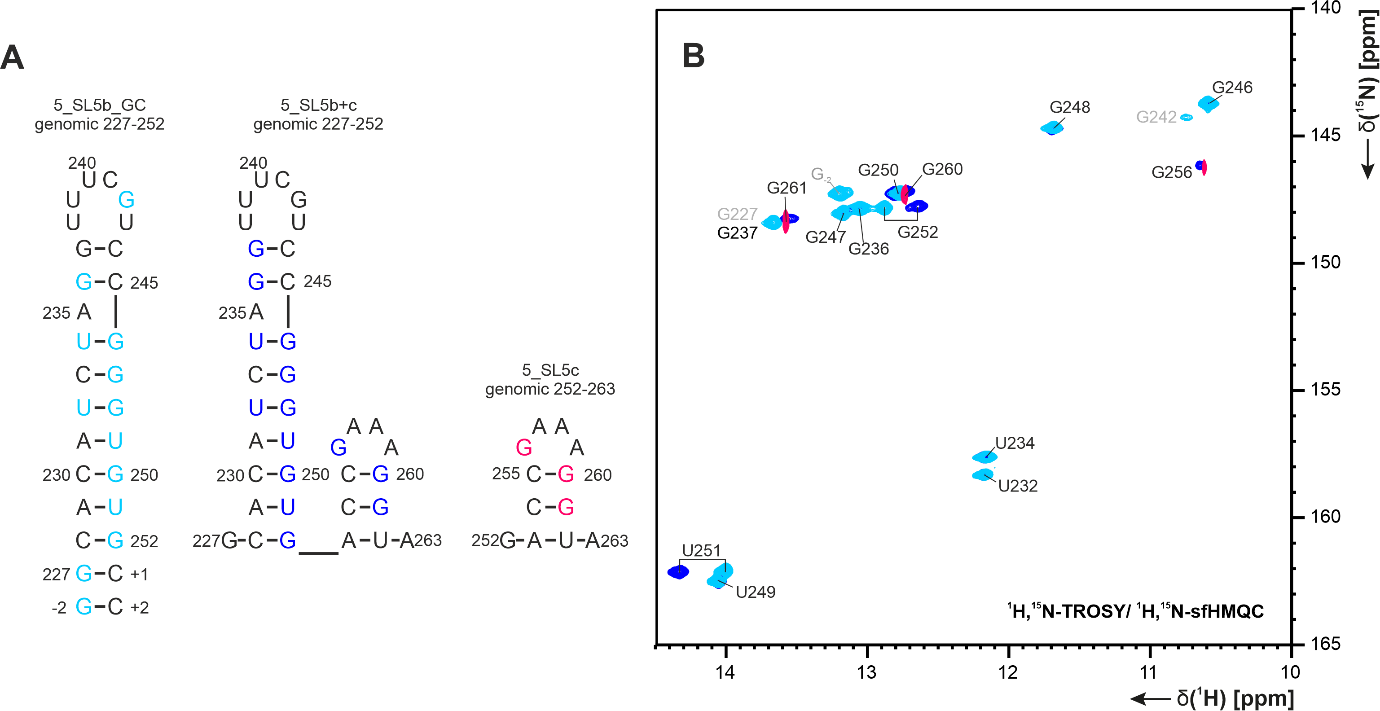
**

**SI Figure 1: A** Predicted secondary structures of RNAs used for the divide-and-conquer approach of element SL5b+c. **B** Comparison of ^1^H,^15^N-imino regions of SL5b+c to SL5b_GC and SL5c to verify consistent secondary structure by chemical shift similarity of sub-elements at 283 K (for experimental data see **Table 1 I, SI Table 1 I, and SI Table 2 I**).

**
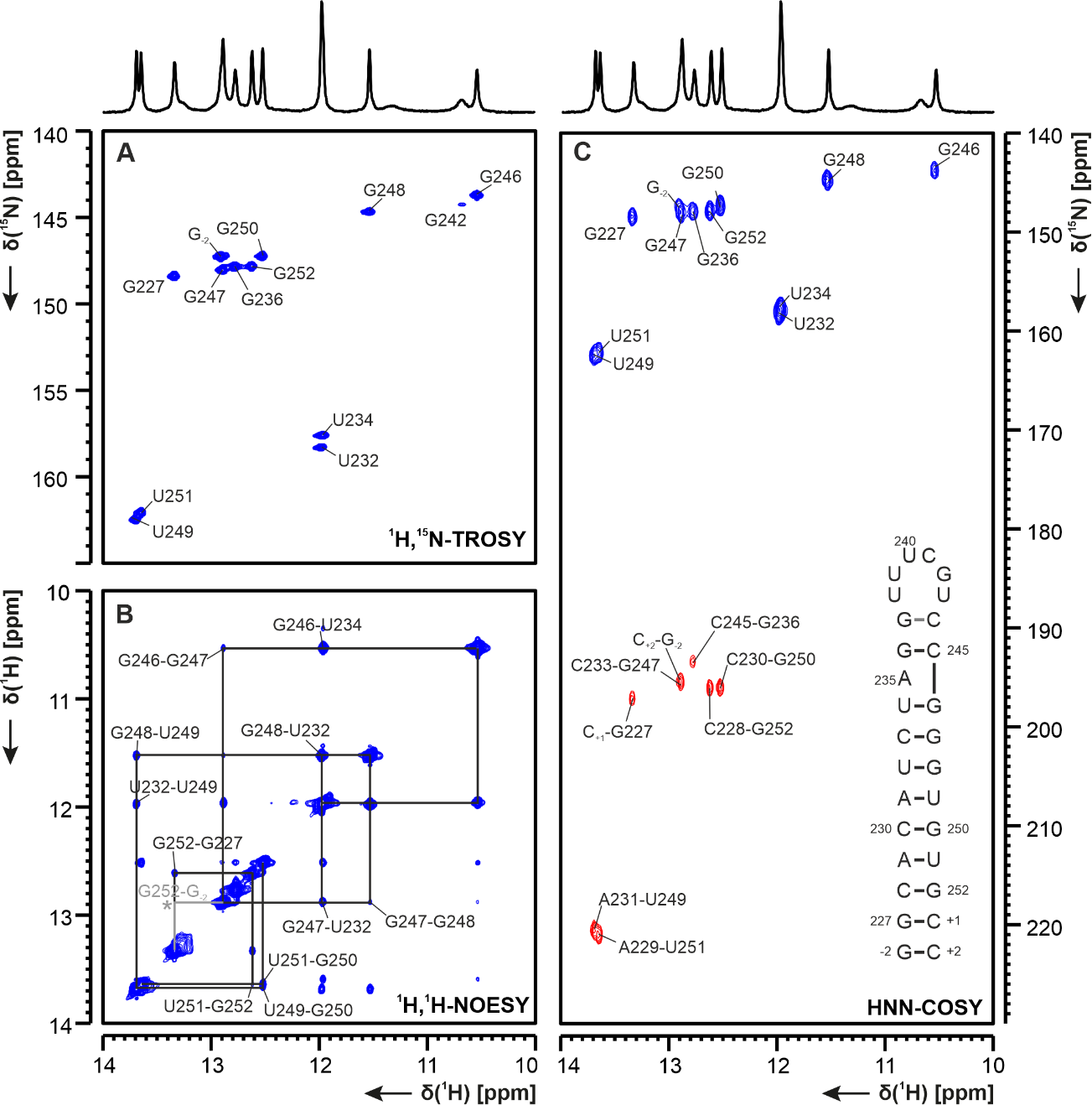
**

**SI Figure 2** **A** ^1^H,15N-TROSY, **B** ^1^H,^1^H-NOESY and **C** HNN-COSY spectra for imino-proton correlation of SL5b_GC at 283K. Positive contours are given in blue, negative contours in red. The imino-proton correlations are annotated using the genomic numbering. Imino-proton correlations in **B** of closing base pairs are shown in grey, * denote crosspeak visible at lower contour setting. Included in **C** is the overall experimentally observed secondary structure of SL5b_GC with genomic numbering. Additional closing base pairs are annotated with ‘±x’. For experimental data see **SI Table 1** I-III.


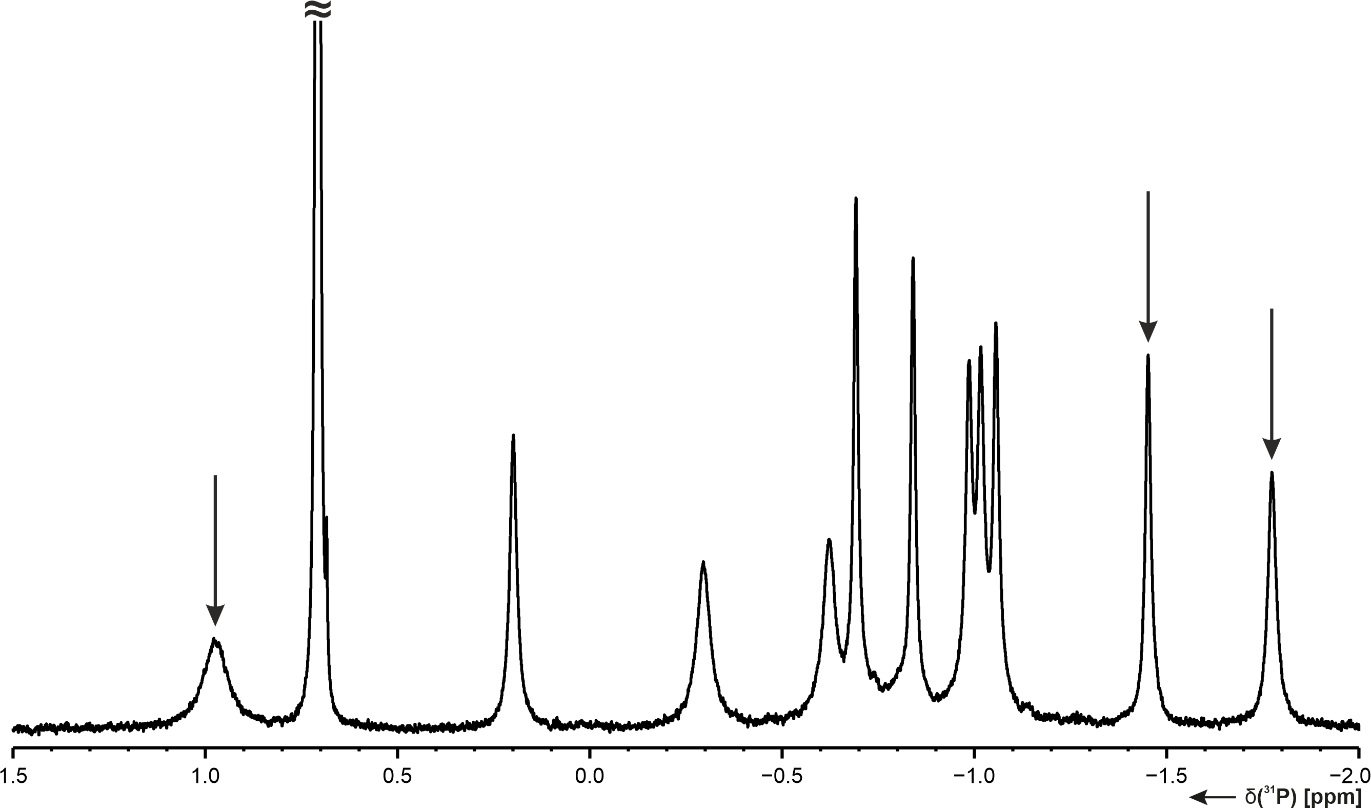


**SI Figure 3** ^31^P 1D spectrum of the SL5c GAAA tetraloop at 298 K in buffer (100% D_2_O). Highlighted by arrows are typical GAAA tetraloop shift patterns for ^31^P as described in the literature (Legault & Pardi, 1994).


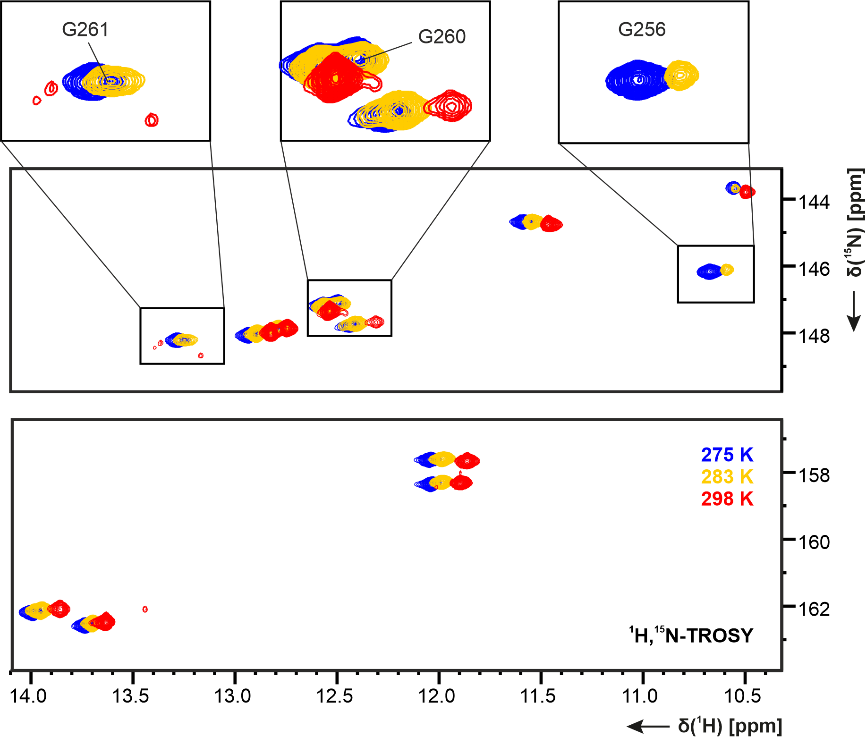


**SI Figure 4** ^1^H,^15^N-TROSY-based comparison of SL5b+c measured between 275 K and 298 K showing temperature response of the 15 imino signals (experimental information of spectra in **Table 1 I**). The signals of SL5c G256, G260, and G261 are not detectable at 298 K.

**SI Table 1** List of NMR experiments for SL5b_GC conducted at BMRZ at temperatures a: 278 K, b: 283 K or c: 298 K. Spectra were recorded in NMR buffer with A: 95% H_2_O/ 5% D_2_O or B: 100% D_2_O. Experimental parameters and experiment-specific parameters are given. ns = number of scans, sw = spectral width, aq = acquisition time, o1/2/3 = carrier frequencies on channels 1/2/3, rel. delay = relaxation delay, CT = constant time, JR = jump-return, fw = forward.

| **#** | **NMR experiment** | **Experimental parameters** | **Characteristic parameters** |
| --- | --- | --- | --- |
| **I** | **^1^H,^15^N-TROSY^BMRZ^** | **A** a 700 MHz, ns: 16, sw(f2): 21.0 ppm, sw(f1): 24.6 ppm, aq(f2): 68.6 ms, aq(f1): 73.1 ms, o1(^1^H): 4.7 ppm, o2(^13^C): 101 ppm, o3(^15^N): 153 ppm, rel. delay: 0.3 s, time: 30 min  **A** b 600 MHz, ns: 8, sw(f2): 24.0 ppm, sw(f1): 25.3 ppm, aq(f2):62.3 ms, aq(f1): 83.2 ms, o1(^1^H): 4.7 ppm, o2(^13^C): 101 ppm, o3(^15^N): 153 ppm, rel. delay: 0.3 s, time: 15 min  **A** c 800 MHz, ns: 16, sw(f2): 21.0 ppm, sw(f1): 24.6 ppm, aq(f2): xx ms, aq(f1): 60.0 ms, o1(^1^H): 4.7 ppm, o2(^13^C): 101 ppm, o3(^15^N): 153 ppm, rel. delay: 0.3 s, time: 1 h |  |
| **II** | **^1^H,^15^N-HNN-COSY^BMRZ^** | **A** b 600 MHz, ns: 16, sw(f3,^1^H): 8.75 ppm, sw(f2,^15^N): 90 ppm, sw(f1,^15^N): 120 ppm, aq(f3): 62.7 ms, aq(f2): 0.09 ms, aq(f1): 13.1 ms, o1(^1^H): 4.7 ppm, o2(^13^C): 185 ppm, o3(^15^N): 185 ppm, rel. delay: 0.3 s, time: 2 h  **A** c 600 MHz, ns: 64, sw(f3,^1^H): 19.8 ppm, sw(f2,^15^N): 30 ppm, sw(f1,^15^N): 100 ppm, aq(f3): 105 ms, aq(f2): 0.03 ms, aq(f1): 31.7 ms, o1(^1^H): 4.7 ppm, o2(^13^C): 183 ppm, o3(^15^N): 183 ppm, rel. delay: 0.3 s, time: 3 h 35 min |  |
| **III** | **^1^H,^1^H-NOESY^BMRZ^**  jump-return water suppression | **A** b 600 MHz, ns: 128, sw(f2): 20.8 ppm, sw(f1): 11.9 ppm, aq(f2): 31.4 ms, aq(f1): 63.28 ms, o1(^1^H): 4.7 ppm, o2(^13^C): 105 ppm, o3(^15^N): 153 ppm, rel. delay: 1.0 s, time: 19 h 40 min  **A** c 800 MHz, ns: 224, sw(f2): 21.1 ppm, sw(f1): 11.9 ppm, aq(f2): 60.6 ms, aq(f1): 18.5 ms, o1(^1^H): 4.7 ppm, o2(^13^C): 120 ppm, o3(^15^N): 153 ppm, rel. delay: 1.0 s, time: 5 h | **b** NOE mixing time 150 ms, JR-delay 50 µs  **c** NOE mixing time 250 ms, JR-delay 39 µs |
| **IV** | **^1^H,^15^N-HSQC^BMRZ^**  Amino  (Mori et al. 1995) | **A** a 700 MHz, ns: 8, sw(f2): 9.8 ppm, sw(f1): 32.9 ppm, aq(f2): 74.5 ms, aq(f1): 41.1 ms, o1(^1^H): 4.7 ppm, o2(^13^C): 101 ppm, o3(^15^N): 86 ppm, rel. delay: 0.8 s, time: 23 min  **A** b 600 MHz, ns: 32, sw(f2): 10 ppm, sw(f1): 30.8 ppm, aq(f2): 85.2 ms, aq(f1): 68.2 ms, o1(^1^H): 4.7 ppm, o2(^13^C): 101 ppm, o3(^15^N): 86.5 ppm, rel. delay: 0.8 s, time: 2 h | INEPT transfer time 2.2 ms (^1^J_NH_ 110 Hz) |
| **V** | **^1^H,^1^H-xf-NOESY^BMRZ^**    xf: selectively filtering N-bond protons, watergate water suppression  (Ikura and Bax 1992; Piotto et al. 1992; Sklenáŕ et al. 1993) | **A** c 800 MHz, ns: 128, sw(f2): 10 ppm, sw(f1): 6.25 ppm, aq(f2): 64 ms, aq(f1): 49.6 ms, o1(^1^H): 4.7 ppm, o2(^13^C): 115 ppm, o3(^15^N): 86 ppm, rel. delay: 1.0 s, time: 27 min | NOE mixing time 250 ms |
| **VI** | **^1^H,^1^H-TOCSY^BMRZ^**    excitation sculpting water suppression  (Shaka et al. 1988; Hwang and Shaka 1995) | **A** c 800 MHz, ns: 32, sw(f2): 8.75 ppm, sw(f1): 6.25 ppm, aq(f2): 99.8 ms, aq(f1): 51.2 ms, o1(^1^H): 4.7 ppm, o2(^13^C): 101 ppm, o3(^15^N): 86 ppm, rel. delay: 1.0 s, time: 5 h 32 min | CC-TOCSY mixing time 40 ms |
| **VII.1** | **^1^H,^13^C-HSQC^BMRZ^**    aromatic region, H-C2/6/8 | **A** c 800 MHz, ns: 4, sw(f2): 8.3 ppm, sw(f1): 24.0 ppm, aq(f2): 76.8 ms, aq(f1): 39.8 ms, o1(^1^H): 4.7 ppm, o2(^13^C): 143 ppm, o3(^15^N): 150 ppm, rel. delay: 1.0 s, time: 40 min | INEPT transfer time 2.5 ms (^1^J_CH_ 200 Hz), off-resonant Q3 shaped pulse for C5 decoupling at 99 ppm with 15 ppm bandwidth |
| **VII.2** | **^1^H,^13^C-HSQC^BMRZ^**    aromatic region, H-C5 | **A** c 800 MHz, ns: 4, sw(f2): 9.0 ppm, sw(f1): 20.0 ppm, aq(f2): 79.8 ms, aq(f1): 63.6 ms, o1(^1^H): 4.7 ppm, o2(^13^C): 99 ppm, o3(^15^N): 185 ppm, rel. delay: 1.0 s, time: 40 min | INEPT transfer time 2.8 ms (^1^J_CH_ 180 Hz), off-resonant Q3 shaped pulse for C2/6/8 decoupling at 150 ppm with 40 ppm bandwidth |
| **VII.3** | **^1^H,^13^C-HSQC^BMRZ^**    ribose region, H-C1’ | **A** c 800 MHz, ns: 4, sw(f2): 9.0 ppm, sw(f1): 20.0 ppm, aq(f2): 79.8 ms, aq(f1): 63.6 ms, o1(^1^H): 4.7 ppm, o2(^13^C): 90 ppm, o3(^15^N): 158.5 ppm, rel. delay: 1.0 s, time: 40 min | INEPT transfer time 2.9 ms (^1^J_CH_ 170 Hz), off-resonant Q3 shaped pulse for C2’ decoupling at 72 ppm with 12 ppm bandwidth |
| **VIII** | **^1^H,^13^C-ct-HSQC^BMRZ^**  full  (Vuister and Bax 1992) | **A** c 600 MHz, ns: 8, sw(f2): 8.33 ppm, sw(f1): 37.88 ppm, aq(f2): 102.4 ms, aq(f1): 44.8 ms, o1(^1^H): 4.7 ppm, o2(^13^C): 77 ppm, o3(^15^N): 155 ppm, rel. delay: 1.0 s, time: 1 h 20 min | INEPT transfer time 1.5 ms (^1^J_CH_ 165 Hz), CT period 25 ms (^1^J_CC_ 40 Hz) |
| **IX** | **(H)C(CCN)H^BMRZ^**    imino-to-aromatics  (Piotto et al. 1992; Sklenář et al. 1996) | **A** c 600 MHz, ns: 336, sw(f3): 20.9 ppm, sw(f2): 9.9 ppm, aq(f3): 90.2 ms, aq(f2): 42.6 ms, o1(^1^H): 4.7 ppm, o2(^13^C): 137 ppm, o3(^15^N): 154 ppm, rel. delay: 1.8 s, time: 1 d 33 min | CC-TOCSY mixing time 28 ms |
| **X** | **H(N)CO^BMRZ^**  imino-to-carbon  (Favier and Brutscher 2011; Solyom et al. 2013) | **A** c 600 MHz, ns: 512, sw(f3): 21.0 ppm, sw(f1): 22.1 ppm, aq(f3): 62.6 ms, aq(f1): 19.2 ms, o1(^1^H): 4.7 ppm, o2(^13^C): 159 ppm, o3(^15^N): 153 ppm, rel. delay: 0.3 s, time: 8 h 31 min | NC-INEPT transfer time 18 ms (^1^J_CN_ 28 Hz) |
| **XI** | **3D ^13^C-NOESY-HSQC^BMRZ^**    aromatics and ribose  (Piotto et al. 1992; Sklenáŕ et al. 1993) | **A** c 800 MHz, ns: 16, sw(f3,^1^H): 8.75 ppm, sw(f2,^13^C): 21.06 ppm, sw(f1,^1^H): 6.25 ppm, aq(f3): 73.1 ms, aq(f2): 9.4 ms, aq(f1): 20.0 ms, o1(^1^H): 4.7 ppm, o2(^13^C): 137 ppm, o3(^15^N): 154 ppm, rel. delay: 0.9 s, time: 22 h (NUS) | NOE mixing time 200 ms, HSQC transfer time 1.4 ms (^1^J_CH_ 180 Hz) |
| **XII** | **3D TROSY-HCCH-COSY^BMRZ^**    adenine C2-to-C8  (Simon et al. 2001) | **B** c 600 MHz, ns: 16, sw(f3,^1^H): 8.75 ppm, sw(f2,^13^C): 22.1 ppm, sw(f1,^13^C): 58.47 ppm, aq(f3): 97 ms, aq(f2): 9.5 ms, aq(f1): 7.25 ms, o1(^1^H): 4.7 ppm, o2(^13^C): 142.5 ppm, o3(^15^N): 150 ppm, rel. delay: 1.0 s, time: 1 d 20 h | Bruker standard parameter set |
| **XIII.1** | **3D HCCH-TOCSY^BMRZ^**    ribose C1’-to-C2’  (Kay et al. 1993; Richter et al. 2010) | **A** c 800 MHz, ns: 8, sw(f3,^1^H): 8.56 ppm, sw(f2,^13^C): 9.47 ppm, sw(f1,^13^C): 35.5 ppm, aq(f3): 74.7 ms, aq(f2): 21.0 ms, aq(f1): 8.96 ms, o1(^1^H): 4.7 ppm, o2(^13^C): 76.5 ppm, o3(^15^N): 153 ppm, rel. delay: 1.0 s, time: 1 d 3 h | CC-TOCSY mixing time 5.4 ms |
| **XIII.2** | **3D HCCH-TOCSY^BMRZ^**    ribose C1’-to-C5’  (Kay et al. 1993; Richter et al. 2010) | **A** c 800 MHz, ns: 8, sw(f3,^1^H): 8.56 ppm, sw(f2,^13^C): 9.47 ppm, sw(f1,^13^C): 35.5 ppm, aq(f3): 74.7 ms, aq(f2): 21.0 ms, aq(f1): 11.2 ms, o1(^1^H): 4.7 ppm, o2(^13^C): 76.5 ppm, o3(^15^N): 153 ppm, rel. delay: 0.97 s, time: 1 d 9 h 20 min | CC-TOCSY mixing time 16 ms |
| **XIV** | **3D HCN^BMRZ^**    C6/8-N1/9-C1’  (Sklenář et al. 1993) | **A** c 600 MHz, ns: 32, sw(f3,^1^H): 8.33 ppm, sw(f2,^13^C): 13.25 ppm, sw(f1,^15^N): 41.1 ppm, aq(f3): 102.4 ms, aq(f2): 14.0 ms, aq(f1): 25.6 ms, o1(^1^H): 4.7 ppm, o2(^13^C): 115 ppm, o3(^15^N): 158 ppm, rel. delay: 1.0 s, time: 22 h (NUS) |  |
| **XV** | **3D fw directed HCC-TOCSY-CCH^BMRZ^**    ribose H1’-to-H3’  (Schwalbe et al. 1995; Marino et al. 1995; Glaser et al. 1996) | **A** c 600 MHz, ns: 8, sw(f3,^1^H): 8.33 ppm, sw(f2,^13^C): 38.54 ppm, sw(f1,^1^H): 4.17 ppm, aq(f3): 102.4 ms, aq(f2): 8.25 ms, aq(f1): 35.2 ms, o1(^1^H): 4.7 ppm, o2(^13^C): 77 ppm, o3(^15^N): 155 ppm, rel. delay: 1.0 s, time: 1 d 22 h | CC-TOCSY mixing time 9.2 ms |

**SI Table 2:** List of NMR experiments for SL5c conducted at BMRB at 283 K if not noted otherwise. Spectra were recorded in NMR buffer with A: 95% H_2_O/ 5% D_2_O or B: 100% D_2_O. Experimental parameters and experiment-specific parameters are given. ns = number of scans, sw = spectral width, aq = acquisition time, o1/2/3 = carrier frequencies on channels 1/2/3, rel. delay = relaxation delay, JR = jump-return.

| **#** | **NMR experiment** | **Experimental parameters** | **Characteristic parameters** |
| --- | --- | --- | --- |
| **I** | **^1^H,^15^N-sfHMQC^BMRZ^**  (Schanda and Brutscher 2005) | **A** 600 MHz, ns: 4096, sw(f2): 23.9 ppm, sw(f1): 25.3 ppm, aq(f2): 71.2 ms, aq(f1): 41.5 ms, o1(^1^H): 4.7 ppm, o2(^13^C): 101 ppm, o3(^15^N): 153 ppm, rel. delay: 0.3 s, time: 11.5 h |  |
| **II** | **^1^H,^1^H-NOESY^BMRZ^**  jump-return water suppression | **A** iminos: 800 MHz, ns: 208, sw(f2): 21.0 ppm, sw(f1): 11.9 ppm, aq(f2): 60 ms, aq(f1): 31 ms, o1(^1^H): 4.7 ppm, o2(^13^C): 150 ppm, o3(^15^N): 153 ppm, rel. delay: 1.0 s, time: 18 h  **B** aromatics: 600 MHz, ns: 64, sw(f2): 14 ppm, sw(f1): 6 ppm, aq(f2): 122 ms, aq(f1): 83 ms, o1(^1^H): 4.7 ppm, o2(^13^C): 99 ppm, o3(^15^N): 153 ppm, rel. delay: 1.5 s, time: 19 h 45 min | **A**: NOE mixing time 150 ms, JR-delay 200 µs  **B**: NOE mixing time 150 ms, JR-delay 200 µs |
| **III** | **^1^H,^1^H-TOCSY^BMRZ^**    excitation sculpting water suppression    (Shaka et al. 1988; Hwang and Shaka 1995) | **A** 800 MHz, ns: 32, sw(f2): 9.0 ppm, sw(f1): 6.2 ppm, aq(f2): 71 ms, aq(f1): 38 ms, o1(^1^H): 4.7 ppm, o2(^13^C): 101 ppm, o3(^15^N): 86 ppm, rel. delay: 1.0 s, time: 4 h  **B** 600 MHz, ns: 16, sw(f2): 9.0 ppm, sw(f1): 9.0 ppm, aq(f2): 308 ms, aq(f1): 47 ms, o1(^1^H): 4.7 ppm, rel. delay: 1.5 s, time: 4 h 40 min | **A** CC-TOCSY mixing time 40 ms  **B** CC-TOCSY mixing time 80 ms |
| **IV** | **^1^H,^13^C-HSQC^BMRZ^**    full    (Bodenhausen and Ruben 1980) | **B** 600 MHz, ns: 256, sw(f2): 10.0 ppm, sw(f1): 106 ppm, aq(f2): 85 ms, aq(f1): 14 ms, o1(^1^H): 4.7 ppm, o2(^13^C): 108 ppm, o3(^15^N): 154.5 ppm, rel. delay: 1.0 s, time: 11 h 15 min | INEPT transfer time 1.47 ms (^1^J_CH_ 170 Hz) |

**References**

Bodenhausen G, Ruben DJ (1980) Natural abundance nitrogen-15 NMR by enhanced heteronuclear spectroscopy. Chemical Physics Letters 69:185–189. <https://doi.org/10.1016/0009-2614(80)80041-8>

Favier A, Brutscher B (2011) Recovering lost magnetization: polarization enhancement in biomolecular NMR. J Biomol NMR 49:9–15. <https://doi.org/10.1007/s10858-010-9461-5>

Glaser SJ, Schwalbe H, Marino JP, Griesinger C (1996) Directed TOCSY, a method for selection of directed correlations by optimal combinations of isotropic and longitudinal mixing. J Magn Reson B 112:160–180. <https://doi.org/10.1006/JMRB.1996.0126>

Hwang TL, Shaka AJ (1995) Water Suppression That Works. Excitation Sculpting Using Arbitrary Wave-Forms and Pulsed-Field Gradients. Journal of Magnetic Resonance, Series A 112:275–279. <https://doi.org/10.1006/jmra.1995.1047>

Ikura M, Bax A (1992) Isotope-filtered 2D NMR of a protein-peptide complex: study of a skeletal muscle myosin light chain kinase fragment bound to calmodulin. J. Am. Chem. Soc. 114:2433–2440. <https://doi.org/10.1021/ja00033a019>

Kay LE, Xu GY, Singer AU, Muhandiram DR, Formankay JD (1993) A Gradient-Enhanced HCCH-TOCSY Experiment for Recording Side-Chain 1H and 13C Correlations in H2O Samples of Proteins. J Magn Reson B 101:333–337. <https://doi.org/10.1006/JMRB.1993.1053>

Marino JP, Schwalbe H, Anklin C, Bermel W, Crothers DM, Griesinger C (1995) Sequential correlation of anomeric ribose protons and intervening phosphorus in RNA oligonucleotides by a 1H, 13C, 31P triple resonance experiment: HCP-CCH-TOCSY. J Biomol NMR 5:87–92. <https://doi.org/10.1007/BF00227473>

Mori S, Abeygunawardana C, Johnson MO, van Zijl PC (1995) Improved sensitivity of HSQC spectra of exchanging protons at short interscan delays using a new fast HSQC (FHSQC) detection scheme that avoids water saturation. J Magn Reson B 108:94–98. <https://doi.org/10.1006/JMRB.1995.1109>

Piotto M, Saudek V, Sklenár V (1992) Gradient-tailored excitation for single-quantum NMR spectroscopy of aqueous solutions. J Biomol NMR 2:661–665. <https://doi.org/10.1007/BF02192855>

Richter C, Kovacs H, Buck J, Wacker A, Fürtig B, Bermel W, Schwalbe H (2010) 13C-direct detected NMR experiments for the sequential J-based resonance assignment of RNA oligonucleotides. J Biomol NMR 47:259–269. <https://doi.org/10.1007/S10858-010-9429-5>

Schanda P, Brutscher B (2005) Very fast two-dimensional NMR spectroscopy for real-time investigation of dynamic events in proteins on the time scale of seconds. J Am Chem Soc 127:8014–8015. <https://doi.org/10.1021/ja051306e>

Schwalbe H, Marino JP, Glaser SJ, Griesinger C (1995) Measurement of H,H-Coupling Constants Associated with .nu.1, .nu. 2, and .nu.3 in Uniformly 13C-Labeled RNA by HCC-TOCSY-CCH-E.COSY. J. Am. Chem. Soc. 117:7251–7252. <https://doi.org/10.1021/ja00132a028>

Shaka AJ, Lee CJ, Pines A (1988) Iterative schemes for bilinear operators; application to spin decoupling. Journal of Magnetic Resonance (1969) 77:274–293. <https://doi.org/10.1016/0022-2364(88)90178-3>

Simon B, Zanier K, Sattler M (2001) A TROSY relayed HCCH-COSY experiment for correlating adenine H2/H8 resonances in uniformly 13C-labeled RNA molecules. J Biomol NMR 20:173–176. <https://doi.org/10.1023/A:1011214914452>

Sklenár V, Peterson RD, Rejante MR, Feigon J (1993) Two- and three-dimensional HCN experiments for correlating base and sugar resonances in 15N,13C-labeled RNA oligonucleotides. J Biomol NMR 3:721–727. <https://doi.org/10.1007/BF00198375>

Sklenár V, Dieckmann T, Butcher SE, Feigon J (1996) Through-bond correlation of imino and aromatic resonances in 13C-, 15N-labeled RNA via heteronuclear TOCSY. J Biomol NMR 7:83–87. <https://doi.org/10.1007/BF00190460>

Solyom Z, Schwarten M, Geist L, Konrat R, Willbold D, Brutscher B (2013) BEST-TROSY experiments for time-efficient sequential resonance assignment of large disordered proteins. J Biomol NMR 55:311–321. <https://doi.org/10.1007/S10858-013-9715-0>
